# Value of three-dimensional imaging in tricuspid valve stenosis: a case series

**DOI:** 10.1186/s43044-025-00672-w

**Published:** 2025-07-30

**Authors:** Mahmoud Abdelnabi, Abdallah Almaghraby, Ramzi Ibrahim, Hoda Abdelgawad

**Affiliations:** 1 Department of Cardiovascular Medicine, Mayo Clinic, Phoenix, Arizona, USA., Phoenix, United States; 2 Cardiology Department, Ibrahim Bin Hamad Obaidallah Hospital, Ras Al Khaimah, UAE., Ras Al Khaimah, United Arab Emirates; 3 Department of Cardiology, King’s College Hospital NHS Trust, London, UK., London, United Kingdom; 4 Department of Cardiology, Faculty of Medicine, Alexandria University, Alexandria, Egypt., Alexandria, Egypt

**Keywords:** Two-dimensional echocardiography (2DE), Three-dimensional echocardiography (3DE), Tricuspid valve, Rheumatic heart disease, Tricuspid stenosis, Commissural fusion, Tricuspid valve area

## Abstract

**Background:**

Rheumatic tricuspid stenosis (TS) is a rare and easily missed clinical finding until late stages. Two-dimensional echocardiography (2DE) can assess the leaflet thickening and transvalvular gradients, which may be misleading in the presence of significant TR. Moreover, it lacks en face views of the tricuspid valve (TV), making the diagnosis of TS challenging. Meanwhile, three-dimensional echocardiography (3DE) effectively visualizes commissural fusion and sub-valvular thickening, enabling accurate tracing of the orifice area.

**Case presentation:**

The authors present a case series discussing rheumatic and non-rheumatic TV stenosis to emphasize the role of 3DE in identifying the key findings in rheumatic stenotic TV and distinguishing them from other non-rheumatic etiologies.

**Conclusions:**

This case series demonstrated that 2DE has limitations in diagnosing TV stenosis, whereas 3DE provides a clear view of features such as commissural fusion and chordal thickening. Therefore, 3DE is essential in addition to 2DE for improved imaging of TV diseases, allowing accurate tracing of the orifice area, regardless of regurgitation.

**Supplementary Information:**

The online version contains supplementary material available at 10.1186/s43044-025-00672-w.

## Background

Three-dimensional echocardiography (3DE) has emerged as a leading technique for imaging rheumatic valves, especially the tricuspid valve. Its popularity has grown due to its superior ability to assess the tricuspid valve's complex anatomy and morphology compared to two-dimensional echocardiography (2DE) [1]. The unequal size, thinness, and variable number of the tricuspid valve leaflets present challenges for 2D imaging. Notably, 3DE enables direct visualization of the tricuspid leaflets (septal, anterior, and posterior) from both atrial and ventricular perspectives, including the commissures and sub-valvular apparatus. Using the apical 4-chamber, RV inflow or short-axis view, we have acquired 3D zoom volume of the TV including a part of either aortic valve or the septum for proper anatomical orientation. Further analysis using the multiplanar reformatting was performed for precise orifice area planimetry [2, 3].

## Case presentation

### Case 1

**Fig. 1:**
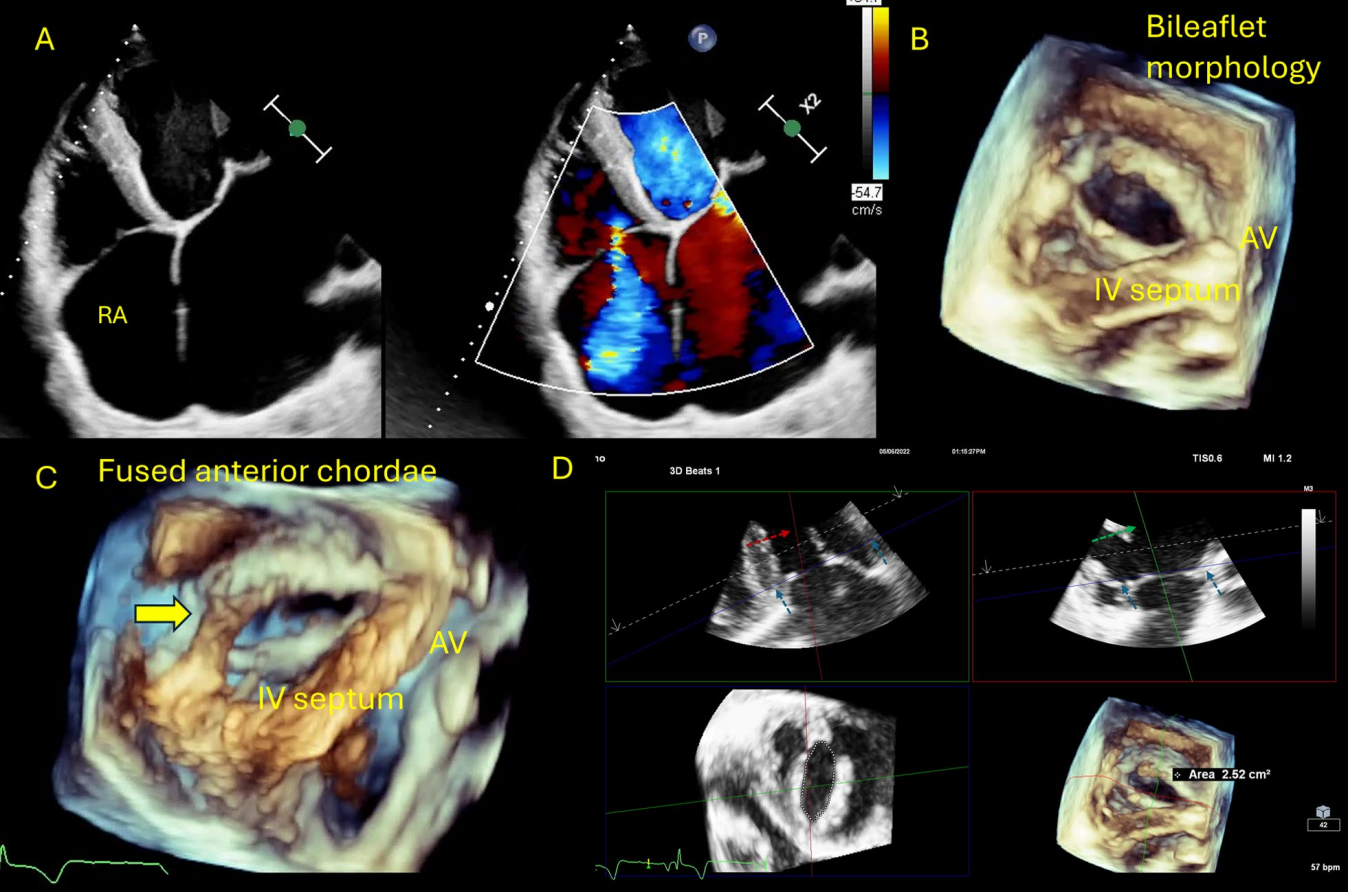
2D/3D imaging of the tricuspid valve; **A** 2D RV-focused apical view showed moderate regurgitation; **B** 3D imaging of the TV from the RV perspective showed type II morphology with fused anteroposterior commissure. **C** Thickened, shortened, and fused chordae of the anterior papillary muscle. **D** MPR-derived valve area planimetry of 2.5 cm^2^

A 45-year-old man presented with progressive shortness of breath and bilateral lower limb swelling, along with fast atrial fibrillation. On examination, a pansystolic murmur was noted over the apex and lower left sternal border, which intensified with inspiration. A 2D transthoracic echocardiogram (TTE) using Philips EPIQ CVx with X5-1 probe revealed rheumatic mitral and aortic valves with severe mitral regurgitation and moderate to severe aortic regurgitation. Parasternal and apical views of the tricuspid valve (TV) showed mildly thickened leaflets and moderate central regurgitation (Fig. 1A). The average mean gradient was 4 mmHg. A 3D acquisition of the TV demonstrated a morphologically bi-leaflet valve (type II, septal, and non-septal configuration) with a fused anteroposterior commissure (Fig. 1B). Multiplanar reconstruction, aligning the slicing planes at the valve ostium in maximum diastole, revealed an area of 2.5 cm^2^ by direct planimetry (Fig. 1D, video 1). Additionally, thickened and fused chordae were clearly visualized (Fig. 1C, video 1), suggestive of rheumatic tricuspid regurgitation without significant stenosis.

### Case 2

**Fig. 2:**
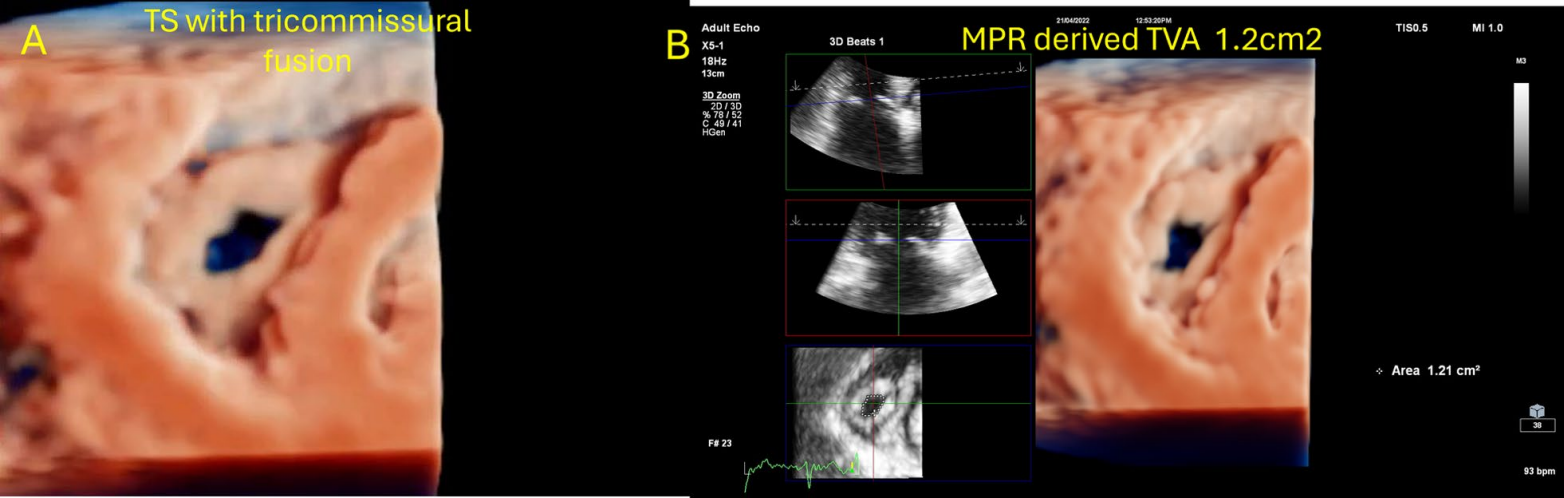
**A** 3D imaging of the tricuspid valve showed markedly thickened leaflets with tri-commissural fusion **B** MPR-derived TVA of 1.2 cm^2^ reporting significant TS

A 43-year-old woman presented for transesophageal echocardiography (TOE) for balloon mitral valvuloplasty. She had a known history of atrial fibrillation and was on warfarin. 2D TTE revealed a typical rheumatic mitral valve with moderate stenosis and moderate aortic stenosis. The TV displayed thickened, doming leaflets with a mean gradient of 12 mmHg, lack of coaptation, and severe regurgitation. Notably, 3D visualization of the tricuspid valve showed type II morphology with restricted septal leaflet motion and fused anteroposterior, anteroseptal, and posteroseptal commissures (Fig. 2A, Video 2), with a traced area of 1.2 cm^2^ suggestive of significant tricuspid stenosis (Fig. 2B).

### Case 3

**Fig. 3:**
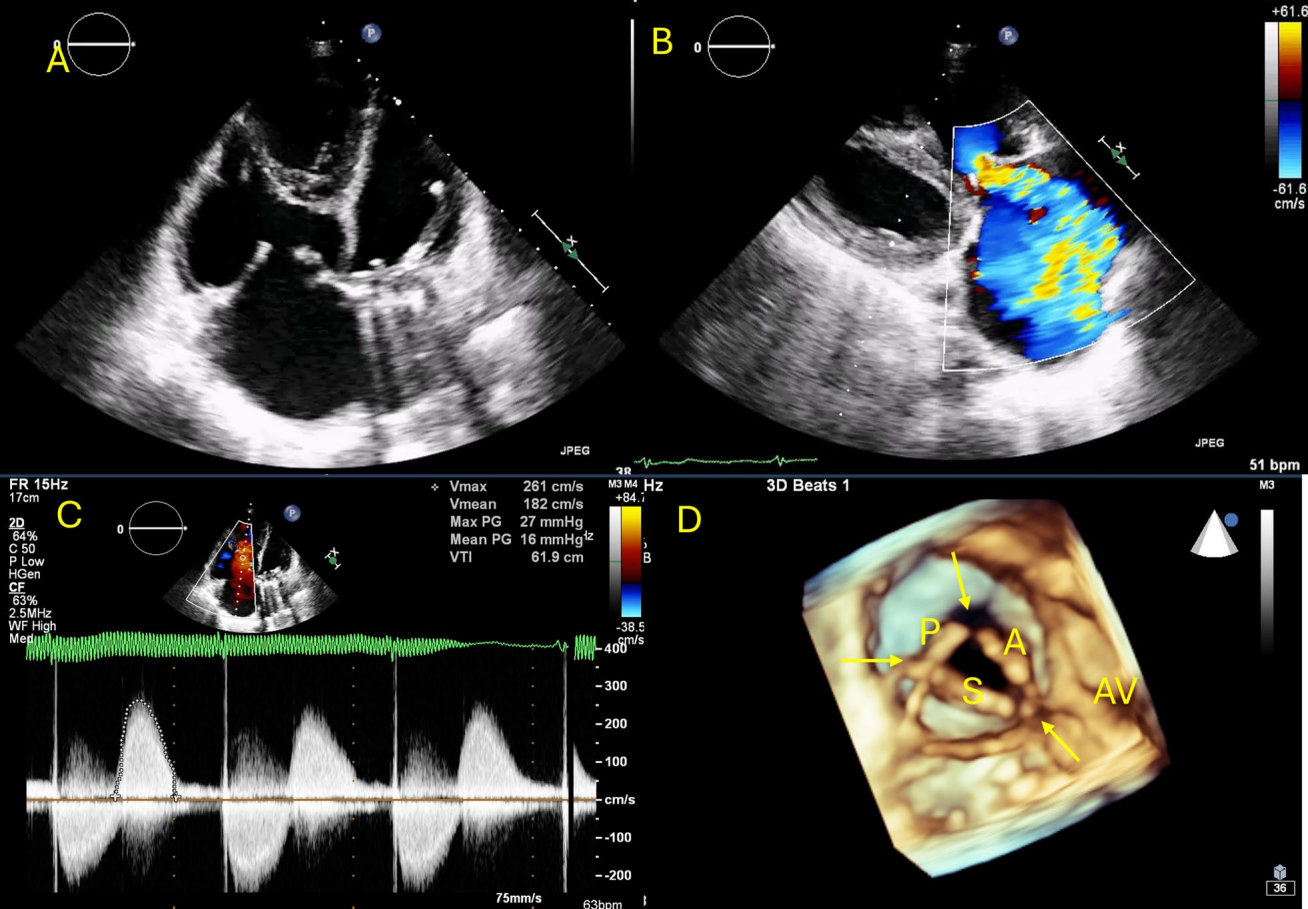
2D/3D imaging of the tricuspid valve in carcinoid heart. **A** RV-focused apical view showed thickened leaflets with reduced excursion and lack of coaptation. **B** Massive tricuspid regurgitation on color Doppler. **C** Mean transvalvular gradient of 16 mmHg. **D** En face visualization of the TV from the RV perspective showing a unique commissural soaring pattern

A 48-year-old woman presented with wheezing, diarrhea, and progressive lower limb swelling, with no history of rheumatic fever. A 2D TTE revealed normal left-sided valves; however, the tricuspid valve had markedly thickened and restricted leaflets, exhibiting a lack of coaptation during systole (Fig. 3A). The mean transvalvular gradient was 16 mmHg. However, massive regurgitation was noted on color Doppler (Fig. 3B and C). From a ventricular perspective, 3D imaging of the tricuspid valve confirmed nearly fixed, non-coapting leaflets with characteristic commissural sparing (Fig. 3D). A traced planimetric area of 1.8 cm^2^ was identified. Based on these findings, we diagnosed carcinoid tricuspid valve disease complicated by massive regurgitation and mild stenosis, and further workup confirmed the presence of carcinoid syndrome.

## Discussion

**Fig. 4:**
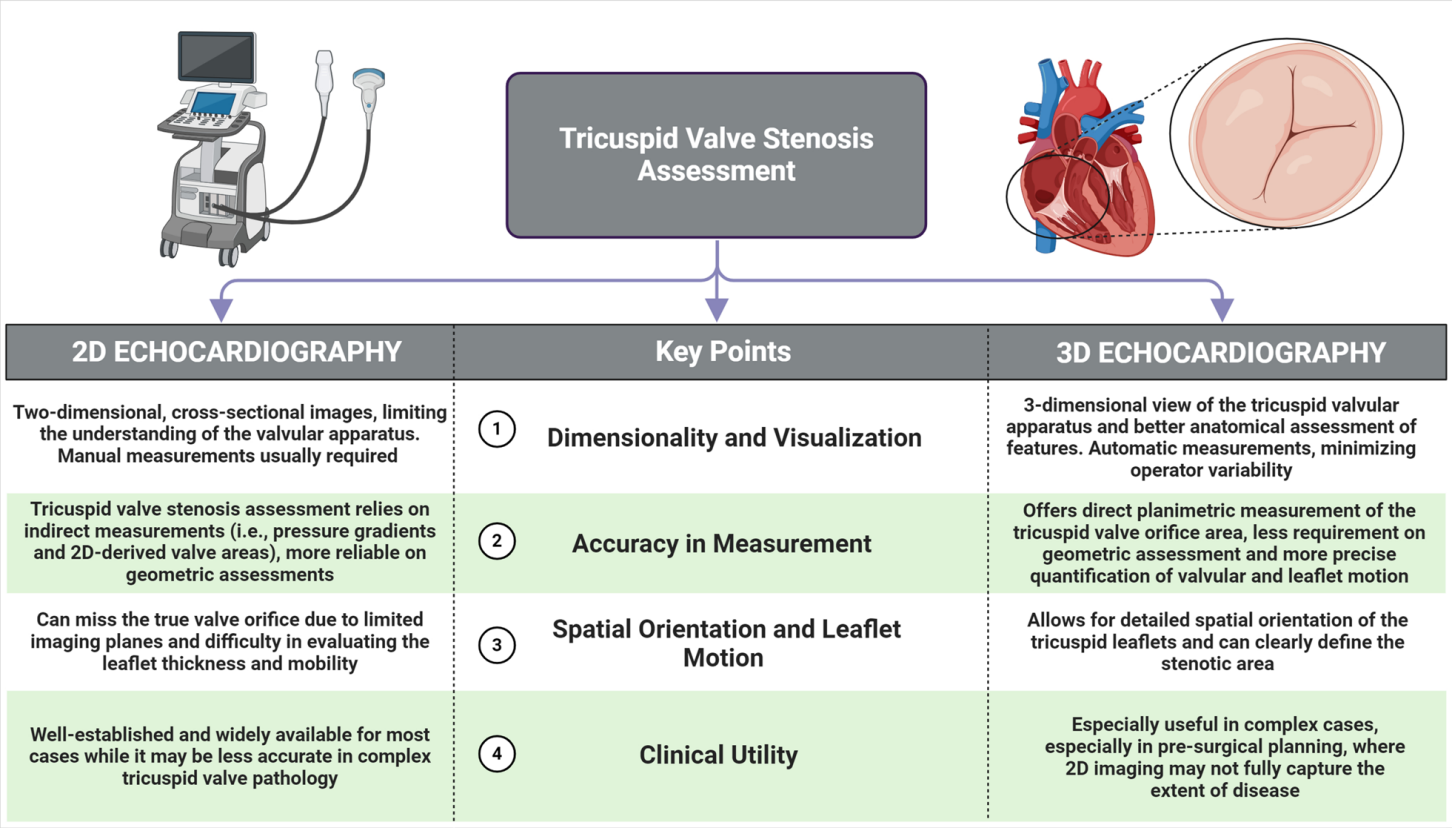
Central illustration highlighting the differences between 2 and 3DE in TV stenosis assessment

Many challenges have limited the 2D echocardiographic assessment of the TV, given the non-planar geometry of the valve orifice, the lack of simultaneous visualization of the three leaflets in a single plane, and the wide range of leaflet numbers. Recently, 3DE has been considered the gold standard for imaging the TV and planning procedural interventions in regurgitant valves [4, 5]. Rheumatic tricuspid valves exhibit unique characteristics that help differentiate them from other primary valve diseases, such as carcinoid valves, which often display commissural sparing features. The first two cases showed mildly thickened leaflets by 2DE. However, more key features were clearly recognized on 3D images, especially commissural fusion at one commissure (anteroposterior commissure in case 1) and three commissures (anteroposterior, anteroseptal, and posteroseptal commissures as in case 2), giving a morphologically bi-leaflet valve. Moreover, thickened, fused, and shortened chordae tendinae were described. Interestingly, the third case shared some morphological findings, such as thickened leaflets with a reduced excursion, with rheumatic valves. Still, the presence of open, non-fused commissures was a clear-cut point that precluded the rheumatic diagnosis. Thus, the diagnosis of carcinoid tricuspid valve was reached in concordance with the clinical and imaging workup. One more advantage is the accurate planimetry of the orifice area, first, by acquiring a high frame rate, single beat, or multi-beat 3D volume of the tricuspid valve, including part of the septum and the aortic valve; second, by selecting the multiplanar reconstruction option and adjusting the slicing planes at the tips of the leaflets and perpendicular to the orifice in the two orthogonal planes in diastole and last, short-axis images of the valve orifice will be created in the third image with the ability of direct area tracing. This feature was extremely useful in the three cases to identify the stenotic degree while being load independent in the presence of hemodynamically significant tricuspid regurgitation or atrial fibrillation (differences between 2 and 3DE are highlighted in Fig. 4). A useful practical workflow of 3DE assessment of TV involves several key steps:

**1. Patient Positioning and Probe Selection:** Use standard echocardiographic windows (apical, parasternal, subcostal) for transthoracic 3DE or midesophageal positions for transesophageal 3DE. Optimize patient position to minimize lung artifact and maximize right heart visualization [3, 6].

**2.** **Data Acquisition:** Acquire a full-volume 3D dataset using ECG gating, typically over 4–7 cardiac cycles to balance spatial and temporal resolution. For focused assessment, use a narrow-angled, single-beat 3D zoom mode to improve resolution of the TV apparatus. The American Society of Echocardiography recommends optimizing 2D images before 3D acquisition to ensure high-quality datasets [3, 6].

**3. Orientation and Display:** Reconstruct the TV in an en face view from the right atrial or right ventricular perspective. The septal leaflet should be positioned at the 6 o’clock position for consistency, as recommended by the American Society of Echocardiography and the European Association of Echocardiography [3, 6].

**4. Multiplanar Reconstruction (MPR):** Use MPR to align orthogonal planes through the annulus and leaflets, allowing precise measurement of annular dimensions, leaflet morphology, and coaptation. This is essential for accurate quantification, as 2D measurements systematically underestimate annular size and do not account for nonplanarity [3, 6].

**5. Quantitative Measurements:** Measure annular area, perimeter, major and minor axes, and leaflet tethering. Dedicated 3D software that accounts for annular nonplanarity is preferred for accuracy. For regurgitation, use color Doppler 3DE to localize jets and planimeter the vena contracta area [3, 6].

## Conclusions

This case series highlights the limitations of 2DE in diagnosing rheumatic tricuspid valve disease compared to the superior capabilities of 3DE. The presented cases demonstrated how 3D imaging enables precise visualization of key rheumatic features such as commissural fusion and chordal thickening, facilitating more accurate diagnosis and therapeutic planning. Therefore, 3DE should be considered essential for comprehensive assessment and management of rheumatic and non-rheumatic tricuspid valve disease alongside 2D imaging techniques.

## Supplementary Information

Additional file 1.

Additional file 2.

Additional file 3.

## Data Availability

No datasets were generated or analysed during the current study.

## Abbreviations

2DE: Two-dimensional echocardiography
3DE: Three-dimensional echocardiography
TV: Tricuspid valve
TS: Tricuspid Stenosis
TR: Tricuspid regurgitation
TTE: Transthoracic echocardiogram
TOE: Transesophageal echocardiography
RV: Right ventricle/right ventricular
MPR: Multiplanar reconstruction
cm^2^: Square centimeters
