# Supplementary material for: Value of three-dimensional imaging in tricuspid valve stenosis: a case series
Source: Egypt Heart J. 2025 Jul 30;77:76. doi: 10.1186/s43044-025-00672-w (PMC12311065; doi:10.1186/s43044-025-00672-w)
Supplement: Supplementary file 3 — Additional file 3. [file 43044_2025_672_MOESM3_ESM.docx]

**Video 1:** 3D TV imaging from the RV perspective with bi-leaflet morphology, commissural fusion, and shortened sub-valvular chordae.

**Video 2:** 3D TV imaging from the RV perspective with leaflet morphology, restricted leaflets’ motion, and tri-commissural fusion.
